# Role of *cis*-Monounsaturated Fatty Acids in the Prevention of Coronary Heart Disease

**DOI:** 10.1007/s11883-016-0597-y

**Published:** 2016-05-24

**Authors:** Peter J. Joris, Ronald P. Mensink

**Affiliations:** Department of Human Biology, NUTRIM School of Nutrition and Translational Research in Metabolism, Maastricht University Medical Center, PO Box 616, Maastricht, 6200 MD The Netherlands

**Keywords:** Monounsaturated fatty acids, Oleic acid, Risk markers, Coronary heart disease, Cardiovascular disease

## Abstract

The effects of *cis*-monounsaturated fatty acids (*cis*-MUFAs) on the risk of coronary heart disease (CHD) and on CHD mortality are not clear. Also, dietary recommendations for *cis*-MUFA as derived by various organizations are not in agreement. Earlier studies have mainly focused on the effects of *cis*-MUFA on serum lipids and lipoproteins. More recent studies, however, have also addressed effects of *cis*-MUFA on other non-traditional CHD risk markers such as vascular function markers, postprandial vascular function, and energy intake and metabolism. Although well-designed randomized controlled trials with CHD events as endpoints are missing, several large prospective cohort studies have recently been published on the relationship between *cis*-MUFA and CHD risk. The aim of this paper is to review these new studies that have been published in the last 3 years on the effects of *cis*-MUFA on cardiovascular risk markers and CHD.

## Introduction

**Fig. 1:**
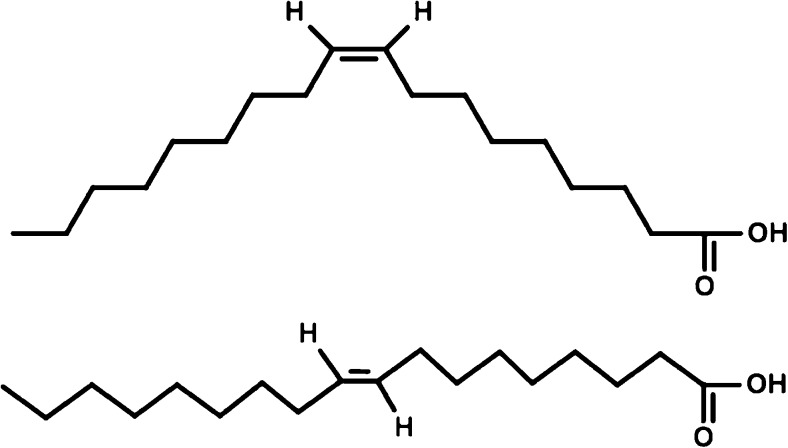
Monounsaturated fatty acids (MUFAs) are chemically classified as fatty acids that have one double bond in the carbon chain. In the *cis*-configuration, the hydrogen atoms attached to the double bond point into the same direction (*top*: oleic acid, a *cis*-MUFA with 18 carbon atoms), while in the *trans*-configuration, the hydrogen atoms are located on opposite sides (*bottom*: elaidic acid, a *trans*-MUFA with 18 carbon atoms)

Optimizing dietary fatty-acid intake is an integral part of dietary guidelines to prevent coronary heart disease (CHD) [1]. In fact, unequivocal evidence exists that eliminating *trans*-fatty acids from hydrogenated oils in the diet lowers the prevalence of CHD [2]. A decrease in the intake of saturated fatty acids (SFA) is also recommended, but in this respect, the type of macronutrient that replaces SFA is important. Replacement of SFA by carbohydrates from refined starches/added sugars may not decrease CHD risk, while replacement by carbohydrates from whole grains or *cis*-polyunsaturated fatty acid (*cis*-PUFA) does [3•, 4]. The effects of *cis*-monounsaturated fatty acids (MUFA; Fig. 1) on CHD risk and CHD mortality are however less clear [5]. Also, dietary recommendations as derived by various health agencies for *trans*-fatty acids, SFA, and *cis*-PUFA are generally more in agreement than those for *cis*-MUFA. For example, no specific dietary reference values for *cis*-MUFA have been formulated in the very recent 2015–2020 Dietary Guidelines for Americans [6], while other organizations have set reference values for *cis*-MUFA [7]. In 2012, Schwingshackl and Hoffmann have reviewed the available evidence from systemic reviews and meta-analyses regarding *cis*-MUFA intake and cardiovascular risk [8]. It was concluded that there was no clear justification to formulate specific dietary recommendations for *cis*-MUFA for the primary and secondary prevention of cardiovascular disease (CVD). On the other hand, as no harmful effects of *cis*-MUFA-rich diets are known, more longer term intervention studies were suggested to clarify potential benefits of *cis*-MUFA-rich diets. Since then, several new studies have been published during the last 3 years on the relationship between *cis*-MUFAs and cardiovascular risk markers or CHD, which will be discussed in the present review.

## Food Sources Of *cis*-Monounsaturated Fatty Acids

Worldwide, the mean intake of *cis*-MUFA ranges from 3.5 % of total energy in certain regions of China to about 22 % in Greece. Oleic acid (18:1; *n*-9) is the predominant *cis*-MUFA, accounting for more than 92 % of all MUFAs consumed. Other *cis*-MUFAs are also present in the diet, but only in very small amounts (Table 1). Except for erucic acid (22:1; *n*-9), their health effects have hardly been studied. Animal studies have suggested that erucic acid at high intakes may cause myocardial lipidosis due to poor mitochondrial β-oxidation. However, rapeseed oil varieties low in erucic acid are nowadays part of the food chain. Therefore, *cis*-MUFA intakes in the studies discussed mainly refer to intakes of oleic acid.

**Table 1:** Overview of different types of *cis*-monounsaturated fatty acids

*cis*-MUFA	Food sources
Caproleic acid (10:1)	Ruminant fats
Lauroleic acid (12:1; *n*-3)	Ruminant fats
Myristoleic acid (14:1; *n*-5)	Ruminant fats
Palmitoleic acid (16:1; *n*-7)	Ruminant fats, fats from fish and marine mammals, macadamia oil, sea buckthorn oil and milkweed seed oil
Oleic acid (18:1; *n*-9)	Vegetable oils such as olive oil, mid-oleic sunflower oil and low-erucic acid rapeseed oil, nuts and seeds, avocados, palm oil, and animal fats
Gadoleic acid (20:1; *n*-11)	Fish oils such as ray, shark and cod, and mustard oil
Erucic acid (22:1; *n*-9)	Mustard oil
Nervonic acid (24:1; *n*-9)	Mustard oil, fish oils such as salmon

Oleic acid is the predominant *cis*-MUFA in the diet, accounting for more than 92 % of all MUFAs consumed

*MUFA* monounsaturated fatty acid

Some vegetable oils, such as olive oil (≈75 %), mid-oleic sunflower oil (≈70 %), and rapeseed oil (≈65 %), consist of more than 50 % of *cis*-MUFA, followed by other products such as palm oil, nuts and seeds, avocados, and animal fats [9]. In fact, animal fats are the main source of *cis*-MUFA in a typical American diet, and high *cis*-MUFA and SFA intakes may thus be correlated. The six food groups with the highest contribution to total MUFA intake among US adults based on the National Health and Nutrition Examination Survey (NHANES) 2003–2006 were other fats and oils (10.5 %), beef (9.2 %), cakes/cookies/quick bread/pastry/pie (8.9 %), frankfurters/sausages/luncheon meats (7.5 %), cheese (6.6 %), and margarine and butter (6.4 %) [10].

## *cis*-Monounsaturated Fatty Acid and Cardiovascular Risk Markers

*cis*-MUFA may affect cardiovascular health through effects on a wide variety of markers associated with CHD [11]. This paragraph reviews current evidence on the effects of *cis*-MUFA on serum lipids and lipoproteins, vascular function markers, postprandial vascular function, and energy intake and metabolism.

### Serum Lipids and Lipoproteins

Data from the many earlier well-controlled randomized intervention studies have shown that *cis*-MUFA has a favorable effect on the serum lipoprotein profile. In studies that replaced fats or oils high in SFA with oils rich in *cis*-MUFA, significant decreases were found in serum concentrations of total cholesterol, LDL-cholesterol, and apoB100 and the total to HDL-cholesterol ratio. Serum HDL-cholesterol, apo-AI, and triacylglycerol concentrations hardly changed. Overall, the effects of oils rich in *cis*-PUFA were slightly more favorable than those of oils rich in *cis*-MUFA, especially on LDL-cholesterol and the total to HDL-cholesterol ratio. In these studies, the diets were enriched with *cis*-MUFA from different sources such as olive oil, high-oleic acid sunflower oil, high-oleic acid safflower oil, and rapeseed oil. More recently, other intervention trials have been carried out specifically focusing on *cis*-MUFA. Gilmore et al. reported that in 17 postmenopausal women, consumption of high-MUFA or low-MUFA ground beef for 6 weeks had comparable effects on the serum lipoprotein profile [12]. However, the difference in *cis*-MUFA intake provided by the two types of ground beef was less than 2 g/day, which may have been too small to observe any effects. In a randomized, double-blind, crossover design with 131 abdominally obese volunteers, five oils with comparable amounts of SFA, but differing in the amounts of oleic acid, linoleic acid, α-linolenic acid, or docosahexaenoic acid (DHA) were consumed for 4-week periods [13••]. Effects on serum LDL-cholesterol, HDL-cholesterol, and triacylglycerol concentrations were comparable, confirming the findings that *cis*-unsaturated fatty acids with 18 carbon atoms have comparable effects on serum lipids. An oil rich in *cis*-MUFA and DHA, however, increased LDL-cholesterol and HDL-cholesterol and lowered serum triacylglycerol concentrations as compared with the other four oils [13••]. In a subset of the population, no evidence was found that consumption of the oils had an adverse effect on LDL proteoglycan binding [14], as observed in animal studies after consumption of oils rich in *cis*-MUFA [15]. As expected, a high-palmitic acid diet increased concentrations of total LDL-cholesterol and HDL-cholesterol and had no effects on serum triacylglycerol as compared with a high-oleic acid diet in 18 healthy subjects [16]. Also, olive oil and rapeseed oil were found to have similar effects on the serum lipoprotein profile [17]. In a 24-week parallel study in individuals with type 2 diabetes, a peanut-enriched American Diabetic Association (ADA) meal plan had similar effects on the serum lipoprotein profile compared with a nut-free ADA meal plan [18]. Dietary SFA and total fat intakes between the two groups were not different. In the Dietary Intervention and Vascular Function (DIVAS) trial, a randomized, single-blind, parallel-group intervention study with 195 adults at moderate CVD risk, three groups of subjects consumed for 16 weeks a diet that was rich in SFA, *cis*-MUFA, or *cis*-PUFA. Except for commercially available foods, specially formulated oils and spreads were used high in *cis*-MUFA (refined olive oil and rapeseed oil) and *cis*-PUFA (safflower oil). The high-SFA group received butter. As compared with the high-SFA groups, serum total and LDL-cholesterol concentrations were reduced to the same extent on the high-MUFA and high-PUFA diets. Effects on serum HDL-cholesterol and triacylglycerol were comparable [19••]. Overall, these recent studies are in line with the earlier studies.

The mechanistic aspects of *cis*-MUFA were examined by Labonté and colleagues [20]. In a randomized parallel study, the effects of exchanging carbohydrates for *cis*-MUFA as part of an experimental portfolio diet on apolipoprotein kinetics were investigated in 16 dyslipidemic subjects. The experimental diets were fed for 4 weeks after a 4-week run-in period. The high-MUFA diet increased apo-AI pool size, mainly due to a reduced apo-AI fractional catabolic rate. LDL apoB100 pool size tended to be reduced on the *cis*-MUFA diet through an increase in LDL apoB100 fractional catabolic rate.

### Vascular Function Markers

The DIVAS study was adequately powered to investigate the long-term impact of replacing SFAs with *cis*-MUFAs on various fasting vascular function markers [19••]. It was found that replacing dietary SFAs with *cis*-MUFAs did not affect fasting flow-mediated vasodilation (FMD) of the brachial artery [19••]. These findings are in agreement with those of Sanders and colleagues, who replaced 5.2 % of energy from dietary SFAs by *cis*-MUFAs for 24 weeks in 121 insulin-resistant men and women [21••]. Furthermore, replacement of SFAs had no effect on arterial stiffness, supporting the earlier findings of Sanders et al. reporting no change in carotid-to-femoral pulse wave velocity (PWV_c-f_) when SFAs were replaced with *cis*-MUFAs [21••]. Of the secondary outcome measures, substitution of dietary SFAs by *cis*-MUFAs significantly reduced circulating E-selectin concentrations by 7.8 %, suggesting an improvement in endothelial activity, while night systolic blood pressure was reduced by 4.9 mm of Hg. As discussed by the authors [19••], the large range of recorded daily activity levels may have masked any effects of the intervention on 24-h or daytime ambulatory blood pressure recordings.

### Postprandial Vascular Function

Postprandial vascular responses were compared between normal-weight and obese men following three isocaloric high-fat challenges differing in fatty-acid composition [22]. For this, 18 normal-weight and 18 obese middle-aged men received in a random order a milkshake providing 95 g of fat, which was either high in SFAs, *cis*-MUFAs, or *n*-*3* long-chain PUFAs. Compared with the SFA and *n*-*3* PUFA shakes, it was found that the *cis*-MUFA milkshake resulted in a more pronounced decrease in the augmentation index (AIx)—a measure of the arterial pressure waveform that depends on the tone of peripheral resistance arteries—and blood pressure. Milkshake consumption resulted in increased plasma sICAM1, sICAM3, and sVCAM1 concentrations 4 h postprandially, with no differences in responses between the shakes for these and other (E-selectin and vWF) plasma biomarkers of endothelial function. Lithander et al. [23] compared the effects of a test meal rich in oleic acid with an isoenergetic meal rich in palmitic acid on postprandial vascular function in younger male participants. No differences in PWV_c-f_, AIx or blood pressure were found between the two test meals providing 56 g of fat. Except for subject characteristics, the difference in findings with the study of Esser and colleagues [22] may also be explained by meal composition, because the mixed meal provided by Lithander was higher in carbohydrates and lower in dietary fat (56 g of fat versus 95 g of fat).

### Energy Intake and Metabolism

Mennella and colleagues fed 15 healthy normal-weight subjects in random order 30 mL of high-oleic acid sunflower oil, virgin olive oil, or sunflower oil plus 30 g of bread. After consumption of the oils rich in oleic acid, energy intake was reduced at the subsequent self-chosen lunch, possible related to the increased postprandial concentrations of oleoylethanolamide (OEA), a compound produced by the small intestine that is involved in appetite regulation. However, energy intake over the next 24 h as assessed by food diaries was comparable between the three treatments [24]. Total energy intake as measured during 4 days was lower in a randomized controlled trial (RCT) with 24 healthy elderly overweight participants following consumption of high-oleic acid peanuts and regular peanuts compared with isoenergetic amounts of a high-carbohydrate snack (potato crisps). Despite these reductions in energy intake, no differences in hunger and satiety that were assessed following snack consumption using visual analog scales were observed [25]. Comparable results were reported in another study with high-oleic acid peanuts [26].

In their recent review [27], Krishnan and Cooper concluded that acute-meal studies suggested that diet-induced thermogenesis and fat oxidation were increased on high unsaturated-fat diets as compared with SFA-rich diets. In this respect, no differences were found between MUFA and PUFA. It was further concluded that also long-term dietary interventions may suggest that MUFA-rich diets induced a greater energy expenditure, diet-induced thermogenesis or fat oxidation as compared with SFA-rich diets [27]. More recently, however, Clevenger et al. found no differences in the effects of SFA, MUFA, and PUFA on diet-induced thermogenesis or postprandial substrate oxidation in obese women [28]. On the other hand, Kien and colleagues reported in healthy volunteers a higher rate of fat oxidation during the fasted state on a palmitic-acid rich diet as compared with an oleic-acid rich diet. Diets were provided for 3 weeks [29••]. Overall, there is no unanimous agreement that *cis*-MUFA affects energy metabolism compared with other dietary fatty acids. The few short-term studies that used peanuts are more consistent in this respect and effects may therefore not necessarily be related to *cis*-MUFA intake. Also, it is not known if the effects are sustained on the longer term and are ultimately translated into a lower body weight and an improved cardiometabolic profile.

## *cis*-Monounsaturated Fatty Acid and Cardiovascular Disease

The Mediterranean diet is well known for its high *cis*-MUFA content (16 to 29 % of energy) with olive oil being the predominant source of fat and intakes of SFA below 8 % [30]. The Mediterranean dietary pattern is associated with a reduced cardiovascular risk. Even though not designed to specially evaluate the effects of *cis*-MUFA, the PREvención con DIEeta MEDiterraneá (PREDIMED) trial found that a Mediterranean diet supplemented with extra-virgin olive oil (50 g/day) or mixed nuts (30 g/day) reduced in individuals at high cardiovascular risk the incidence of major cardiovascular events (a composite of myocardial infarction, stroke, or death from CVD causes) as compared with a control diet low in fat [31••]. A meta-analysis of 15 RCTs, that involved more than 50,000 participants, aimed to estimate the effect of replacing dietary SFA for carbohydrates, *cis*-PUFA, *cis*-MUFA, or protein on cardiovascular morbidity and mortality [5]. It was concluded that the effects of *cis*-MUFA were unclear as only one small trial from 1965 was identified [32], in which no effects were observed. In this respect, the results of recently published large prospective cohort studies evaluating the association between *cis*-MUFA intake with the risk of CVD and cardiovascular death may be more informative (Table 2).

**Table 2:** Summary of recent studies assessing the effects of *cis*-MUFAs on cardiovascular disease

Study	Research question	Study design	Results
PREDIMED Study [33•]	Dietary fat intake and risk of total CVD events in a population at high risk of CVD	Prospective cohort study	Dietary SFA for *cis*-MUFA (5 % of energy) HR 0.63 (95 % CI 0.43–0.94)
NHS and the HPFS [3•]	Dietary fat intake and risk of CHD in men and women free of diabetes, CVD, and cancer	Prospective cohort study	Dietary SFA for *cis*-MUFA (5 % of energy) HR 0.85 (95 % CI 0.74–0.97)
ATBC Cancer Prevention Study [34•]	Carbohydrate substitution for dietary fat and risk of CHD in Finnish male smokers	Prospective cohort study	Total carbohydrates for *cis*-MUFA (2 % of energy) RR 0.92 (95 % CI 0.84–0.99)
EPIC Study [35•]	Carbohydrate substitution for dietary fat on mortality risk in patients with type 2 diabetes	Prospective cohort study	Carbohydrates for *cis*-MUFA (5 % of energy) HR 0.87 (95 % CI 0.76–1.00). No effects on CVD mortality risk
EPIC-NL Study [36•]	Dietary fat intake and risk of CHD in a Dutch population	Prospective cohort study	Dietary SFA for *cis*-MUFA (5 % of energy) HR 1.30 (95 % CI 1.02–1.65)

*CVD* cardiovascular disease, *CHD* coronary heart disease, *N/A* not applicable, *SFA* saturated fatty acid, *MUFA* monounsaturated fatty acid, *HR* hazards ratio, *RR* relative risk

Subjects within the PREDIMED trial were also prospectively studied. While the trial was conducted from 2003 to 2010, the present data were based on an expanded follow-up until 2012 [33•]. The mean intake of *cis*-MUFAs (expressed as percentage of energy) was high: 13.4 % in the lowest quintile compared with 26.1 % in the top quintile. After 6 years of follow-up, 336 CVD cases and 414 total deaths were reported. It was found that higher intakes of *cis*-MUFA were inversely associated with CVD, cardiovascular death, and all-cause death. In fact, isocaloric substitution of 5 % of energy from SFAs or *trans*-FAs with *cis*-MUFAs was associated with a 37 and 40 % lower risk of total CVD events. In agreement, an updated analysis of the Nurses’ Health Study (1980–2010; 84,628 US women) and the Health Professional Follow-Up Study (1986–2010; 42,908 US men) showed a reduction in CHD risk when SFA was exchanged for *cis*-MUFA [3•]. In these two large, independent prospective cohorts of men and women, 7667 cases of CHD (4931 nonfatal myocardial infarctions and 2736 CHD deaths) were documented over 24 to 30 years of follow-up. Replacing 5 % of energy from dietary SFA with an equivalent amount of *cis*-MUFA was associated with a 15 % (95 % CI, 3 to 26 %) lower risk of CHD. In a large prospective cohort study carried out within the Alpha-Tocopherol, Beta-Carotene Cancer Prevention Study (ATBC), the associations between glycemic index, substitutions of total, low-, medium-, and high-glycemic-index carbohydrates for dietary fat and the risk of CHD were examined [34•]. During a 19-year follow-up, 4379 CHD cases, including a total of 2377 non-fatal myocardial infarctions and 2002 CHD deaths, were documented among approximately 22,000 middle-aged Finnish male smokers. Substituting carbohydrates for *cis*-MUFAs was associated with a decreased risk. It was estimated that isoenergetic replacements of 2 % of energy from total, low-, or high-glycemic-index carbohydrates for *cis*-MUFAs were associated with 8 % (95 % CI, 1 to 16 %), 9 % (95 % CI 2 to 16 %), and 8 % (95 % CI 1 to 15 %) lower CHD risks. The isocaloric replacement of medium-glycemic-index carbohydrates with *cis*-MUFA tended to decrease the risk of CHD by 7 % (95 % CI 0 to 15 %). Dietary fiber intake modified the association between the replacement of carbohydrates with *cis*-MUFA and the risk of CHD in the stratified analyses. In fact, increasing *cis*-MUFA intake at the expense of carbohydrates was more beneficial among the subjects with a higher fiber intake. In patients with type 2 diabetes, the association between dietary carbohydrate intake and substitution for *cis*-MUFA with cardiovascular and all-cause mortality was also investigated [35•]. A total of 6192 type 2 diabetic patients from 15 cohorts of the European Prospective Investigation into Cancer and Nutrition (EPIC) were included. After a mean follow-up of about 9 years, 268 CVD deaths and 791 total deaths were reported. Substituting 5 % of energy from carbohydrates with *cis*-MUFA may be associated with a lower all-cause mortality risk (HR, 0.87; 95 % CI, 0.76 to 1.00), while no effects were reported for CVD mortality risk.

Surprisingly, a lower CHD risk with a higher intake of SFAs was recently observed that did not depend on the type of substituting macronutrient [36•]. The EPIC-Netherlands (EPIC-NL) cohort included 35,597 Dutch men and women. In this prospective cohort study, a total of 1807 incident CHD cases and 158 CHD deaths (8.7 %) occurred over a median follow-up time of 12 years. After full adjustment, the substitution of 5 % of energy from SFA with *cis*-MUFA was associated with 30 % (95 % CI: 2 % to 65 %) increased risk to develop CHD. These results, however, should be interpreted with caution. As discussed by the authors [36•], residual confounding by unmeasured initiation of cholesterol-lowering therapy during follow-up may explain these findings as adults with high SFA intake have high serum cholesterol concentrations and will become eligible for lipid-lowering therapy during follow-up, which would reduce CHD risk. In addition, the limited variation in dietary SFA intake or the source of SFA may also have attributed to the observed risk differences in this Dutch population.

## Conclusions

In summary, recent studies are in line with the earlier studies showing that *cis*-MUFAs have a favorable effect on the serum lipoprotein profile as compared with a mixture of SFA, while effects are comparable to those of linoleic and α-linolenic acid. Effects on fasting and postprandial vascular function have not been studied extensively and no consistent differences between the various fatty acids are evident. Longer-term studies should address whether products rich in *cis*-MUFA affect energy intake and metabolism compared with other macronutrients. In fact, studies addressing the effects of food sources and matrices are of interest, as this may impact the results. Well-designed RCTs with CVD events as endpoints are lacking. Evidence from large prospective cohort studies regarding effects of *cis*-MUFA on the risk to develop CHD is limited, but several studies do suggest that replacements of SFA or high-glycemic index foods for *cis*-MUFA lowers CHD risk. In this respect, however, *cis*-MUFA is not more beneficial than linoleic acid. More research is thus required on the long-term effects of *cis*-MUFA as compared with other macronutrients on CHD risk markers as well as on clinical endpoints to clarify the potential role of *cis*-MUFAs in the primary and secondary prevention of CHD.
